# Thermo-Fluid Characteristics of High Temperature Molten Salt Flowing in Single-Leaf Type Hollow Paddles

**DOI:** 10.3390/e20080581

**Published:** 2018-08-07

**Authors:** Taha Rajeh, Ping Tu, Hua Lin, Houlei Zhang

**Affiliations:** 1 School of Energy and Power Engineering, Nanjing university of Science and Technology, Nanjing 210094, China; 2 Shenzhen Douwin Technology Co., Ltd., Shenzhen 518116, China; 3 Key Laboratory of Tropical Forest Ecology, Xishuangbanna Tropical Botanical Garden, Chinese Academy of Sciences, Mengla 666303, China

**Keywords:** hollow paddle, molten salt, thermo-fluid, entropy generation rate, trade-off

## Abstract

A single-leaf type paddle heat exchanger with molten salt as the working fluid is a proper option in high temperature heating processes of materials. In this paper, based on computational fluid dynamics (CFD) simulations, we present the thermo-fluid characteristics of high temperature molten salt flowing in single-leaf type hollow paddles in the view of both the first law and the second law of thermodynamics. The results show that the heat transfer rate of the hollow paddles is significantly greater than that of solid paddles. The penalty of the heat transfer enhancement is additional pressure drop and larger total irreversibility (i.e., total entropy generation rate). Increasing the volume of the fluid space helps to enhance the heat transfer, but there exists an upper limit. Hollow paddles are more favorable in heat transfer enhancement for designs with a larger height of the paddles, flow rate of molten salt and material-side heat transfer coefficient. The diameter of the flow holes influences the pressure drop strongly, but their position is not important for heat transfer in the studied range. Other measures of modifying the fluid flow and heat transfer like internal baffles, more flow holes or multiple channels for small fluid volume are further discussed. For few baffles, their effects are limited. More flow holes reduce the pressure drop obviously. For the hollow paddles with small fluid volume, it is possible to increase the heat transfer rate with more fluid channels. The trade-off among fluid flow, heat transfer and mechanical strength is necessary. The thermo-fluid characteristics revealed in this paper will provide guidance for practical designs.

## 1. Introduction

Paddle heat exchangers were first used in drying materials, called paddle dryers [1,2,3,4,5,6,7,8]. In high temperature heating industries (e.g., in the range of 300 to 600 °C), molten salts (e.g., Hitec salt) are commonly used as working fluids for heat transfer. Recently, many researchers have investigated molten salt thermo-fluid characteristics in different geometrical structures. For example, Wu et al. [9] experimentally investigated the turbulent flow and transitional flow of molten Hitec salt in a circular tube. Their results provided different convective heat transfer coefficients of molten Hitec salt. Ferng et al. [10] used the computational fluid dynamics (CFD) technique and the two-equation *k*-*ε* turbulent model to study the thermal-hydraulic characteristics of molten FLiNaK salt in a circular tube. Their numerical simulation results agree with experimental data and general correlations. Also based on CFD simulation, Srivastava et al. [11] investigated the flow and heat transfer characteristics of molten FLiNaK salt in a circular tube for different flow regimes, i.e., laminar, transitional and turbulent flow. Their numerical predictions agreed with that of the available correlations.

Zhang et al. [12] tested the thermal performance of molten salt cavity receivers. They showed that the flow rate does not have a strong effect on the instantaneous efficiency of the receiver in the pro-steady state. Du et al. [13] experimentally studied the heat transfer performance of molten Hitec salt in the shell side of a shell-and-tube heat exchanger with segmental baffles. New Nusselt number correlation in specified turbulent regime was provided. Later, Du et al. [14] further investigated the heat transfer characteristics of molten Hitec salt in the shell side of a shell-and-tube heat exchanger without baffles experimentally and numerically. The two-equation *k*-*ε* turbulent model and Fluent commercial software were used to simulate the convection process. The experimental and numerical results showed good agreement. Lu et al. [15] measured the heat transfer performance of ternary nitrate salt flowing in an annular duct with a cooled wall. Based on experimental data, a new heat transfer correlation in the turbulent regime was proposed. Chen et al. [16], in laminar-transition-turbulent regimes, experimentally investigated the convective heat transfer performance of transversely-grooved tubes with heat transfer salt KNO_3_-NaNO_2_-NaNO_3_ (53-40-7 mol %) as the working fluid. Compared to circular tubes, transversely-grooved tubes show significant heat transfer enhancement. 

Except circular tubes and shell-and-tube heat exchangers, the CFD technique has been used in simulating molten salt flow and heat transfer in other geometrical structures. It has been accepted as an important tool in analysis and design. Based on the Reynolds-averaged Navier–Stokes method, Carasik et al. [17] calculated the turbulent pressure drop of molten salt (FLiBe) flow in an in-line heat exchanger. Several one- and two-equation turbulence models were compared. The standard low Reynolds number *k*-*ε* turbulent model, which provides reasonable predictions, was recommended. Chen et al. [18] simulated the mixed convection heat transfer of Hitec salt in horizontal square tubes. The two-equation RNG *k*-*ε* model was adopted. The comparison of experimental and simulation results showed good agreement. In [19], Hitec salt flow and heat transfer in hollow disc-shaped heaters were simulated by using the standard *k*-*ε* model. The optimal design was suggested based on the simulation results. A brief review of high temperature molten salt heat transfer and heat exchangers was given by Du et al. in [20].

Few studies focused on the fluid flow and heat transfer of molten salt in hollow paddle-shaft structures of paddle heat exchangers. Inspired by constructal law [21], Zhang et al. [22] presented the molten salt fluid flow and heat transfer performance of three two-leaf-type paddle-shaft structures and showed that, in the heat transfer aspect, the open hollow paddle-shaft structure is much better than that of closed hollow paddle-shaft structure and solid paddle-shaft structure. Ji et al. [23] further studied the effects of a guiding plate near the outlet flow hole of a two-leaf-type paddle heat exchanger. Their results show that in the two leaves, the fluid flow and heat transfer are non-symmetrical, and it is possible to enhance the heat transfer and decrease the pressure drop simultaneously. In [24], it was shown that the influence of rotation rate (<10 rpm) on fluid flow and heat transfer is negligible. Compared to those two-leaf-type paddle heat exchangers, the single-leaf type design has a higher heat transfer area and a simpler structure and manufacture. This may be used in non-agglutination or weak-agglutination materials [25]. When the heat transfer coefficients of the two sides (i.e., the working fluid side and the material side) are at the same scale, it is helpful to enhance the heat transfer of the working fluid side. Furthermore, the heat transfer enhancement will also improve the temperature uniformity of the heating surfaces, which is important for heating temperature-sensitive materials. Up to now, to the authors’ knowledge, there are no published data on the thermo-fluid characteristics of high temperature molten salt flowing in single-leaf type hollow paddles.

In this paper, we first describe the geometry of single-leaf type hollow paddles, and then build a CFD model to simulate the fluid flow and heat transfer performance for the structures. Based on the simulation results, we identify the optimal design for heat transfer enhancement. The trade-off in designs is also discussed. This work is the starting point prior to real product design, manufacture and testing.

## 2. Geometry Description

**Figure 1:**
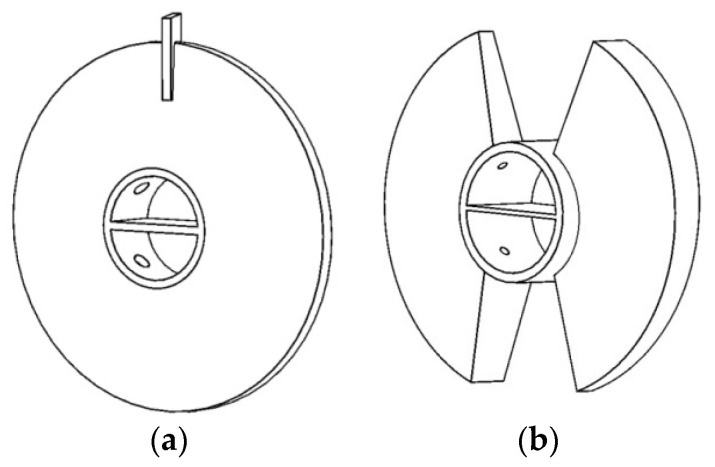
Hollow paddles: (**a**) single-leaf type; and (**b**) two-leaf type.

**Figure 2:**
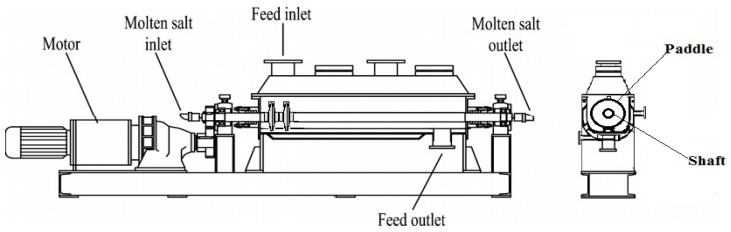
Cross-section of a single-shaft and single-leaf type paddle heat exchanger.

There are two common types of paddles for paddle heat exchangers, i.e., single-leaf type represented in Figure 1a and two-leaf type in Figure 1b. In this study, we only discuss the single-leaf type hollow paddles. Figure 2 shows the cross-section of a heat exchanger with single-leaf type paddles. Under normal working conditions, the working fluid flows in the hollow paddle-shaft structure and heats the shell-side material, which is driven by the rotating paddles. In order to provide sufficient residence time for materials to be heated, the rotation is usually very slow, e.g., less than 10 rpm [24]. The work in [24] has shown that the effect of slow rotation (<10 rpm) is very weak. Therefore, in this paper, we neglect the influence of rotation on flow and heat transfer.

**Figure 3:**
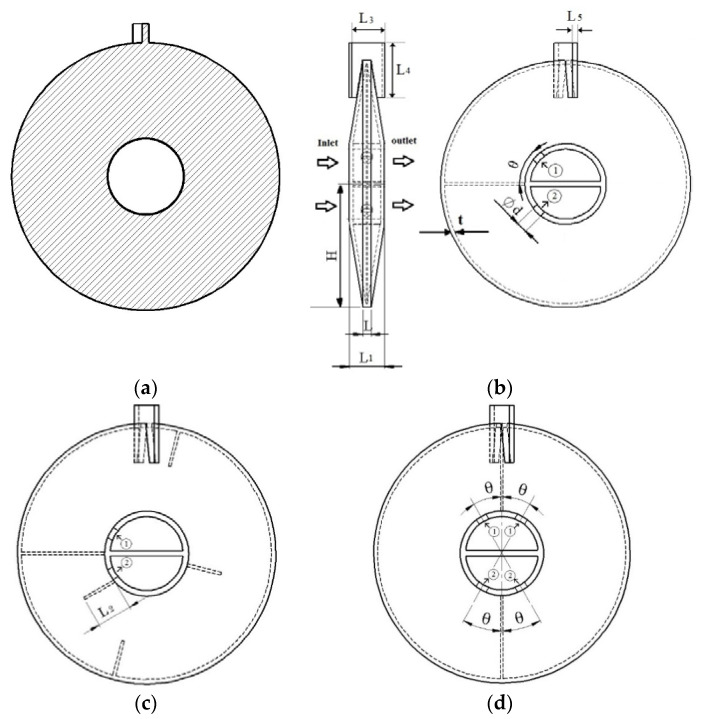
Candidate designs: (**a**) Case I, solid paddle; (**b**) Case II, hollow paddle with two flow holes, (**c**) Case III, hollow paddle with two flow holes and internal baffles; (**d**) Case IV, hollow paddle with four flow holes.

Consider a single-leaf type paddle heat exchanger with Hitec salt (53% KNO_3_, 40% NaNO_2_, 7% NaNO_3_ based on mass fraction) as the working fluid. The overall shaft is composed of some segments. In every segment, there is one paddle for single-leaf type design. We assume that the molten salt is distributed uniformly into all the segments [23], so only one segment is studied. For all shaft-paddle structures to be investigated, the inner and outer diameters of the hollow shaft are the same, so for simplicity, we only discuss the thermo-fluid characteristics in the paddle domain. In order to study the effects of the fluid volume in the hollow paddles, we use the solid paddle design (i.e., zero fluid volume) as the reference. Figure 3 shows the candidate designs: solid paddle and different hollow paddles.

## 3. Numerical Model

In this section, we present a CFD model to simulate the molten salt flow and heat transfer in the paddle domain shown in Figure 3. The basic assumptions for the simulations are: zero rotation rate, steady turbulent forced flow for molten salt, constant temperature and convective heat transfer coefficient of the shell-side material, constant properties of molten salt and the solid and zero radiation of the outer surfaces of the paddle due to the material’s covering.

The three-dimensional mass, momentum and energy equations for molten salt flow without phase change are presented as follows [26]:

(1) $$\frac{\partial }{\partial x_{i}}\left(\rho u_{i}\right)=0,$$

(2) $$\frac{\partial }{\partial x_{j}}(\rho u_{i}u_{j})=-\frac{\partial P}{\partial x_{i}}+\frac{\partial }{\partial x_{j}}\left[\mu \left(\frac{\partial u_{i}}{\partial x_{j}}+\frac{\partial u_{j}}{\partial x_{i}}-\frac{2}{3}\delta_{ij}\frac{\partial u_{l}}{\partial x_{l}}\right)\right]+\frac{\partial }{\partial x_{j}}(-\rho \bar{{u^{\prime }}_{i}{u^{\prime }}_{j}}),$$

(3) $$\frac{\partial }{\partial x_{i}}[u_{i}(\rho e+P)]=\frac{\partial }{\partial x_{j}}\{\left(\lambda +\frac{c_{p}\mu_{t}}{Pr_{t}}\right)\frac{\partial T}{\partial x_{j}}+u_{i}\left[(\mu +\mu_{t})\left(\frac{\partial u_{j}}{\partial x_{i}}+\frac{\partial u_{i}}{\partial x_{j}}\right)-\frac{2}{3}(\mu +\mu_{t})\frac{\partial u_{l}}{\partial x_{l}}\delta_{ij}\right]\},$$

(4) $$e=h-\frac{P}{\rho }+\frac{v^{2}}{2}.$$

For the solid region, the energy equation is:

(5) $$\frac{\partial }{\partial x_{i}}\left(\lambda_{s}\frac{\partial T_{s}}{\partial x_{i}}\right)=0.$$

The turbulent kinetic energy k and its rate of dissipation *ε* are obtained from the transport Equations (6) and (7), and the turbulent viscosity *µ_t_* is calculated by combining *k* and *ε* according to Equation (8):

(6) $$\frac{\partial }{\partial x_{j}}(\rho ku_{j})=\frac{\partial }{\partial x_{j}}\left[\left(\mu +\frac{\mu_{t}}{\sigma_{k}}\right)\frac{\partial k}{\partial x_{j}}\right]-\rho \bar{{u^{\prime }}_{i}{u^{\prime }}_{j}}\frac{\partial u_{j}}{\partial x_{i}}-\rho \varepsilon ,$$

(7) $$\frac{\partial }{\partial x_{j}}(\rho \varepsilon u_{j})=\frac{\partial }{\partial x_{j}}\left[\left(\mu +\frac{\mu_{t}}{\sigma_{\varepsilon }}\right)\frac{\partial \varepsilon }{\partial x_{j}}\right]+C_{1\varepsilon }\frac{\varepsilon }{k}\left(-\rho \bar{{u^{\prime }}_{i}{u^{\prime }}_{j}}\frac{\partial u_{j}}{\partial x_{i}}\right)-C_{2\varepsilon }\rho \frac{\varepsilon^{2}}{k},$$

(8) $$\mu_{t}=\rho C_{\mu }\frac{k^{2}}{\varepsilon },\;$$

In Equation (3), *Pr_t_* = 0.85 and in Equations (6) and (7), the constants adopt the following values: *C*_1_*ε* = 1.44, *C*_2_*ε =* 1.92, *C_µ_* = 0.09, *σ_k_ =* 1, *σ_ε_* = 1.3 [26]. The boundary conditions and the properties of the materials are given in Table 1 and Table 2, respectively. In Figure 3, the following sizes are assumed constant: *L*_3_ = 28 mm, *L*_4_ = 41 mm, *L*_5_ = 4 mm. 

To obtain the flow and temperature fields and the overall thermo-fluid performance (e.g., the pressure drop *ΔP* of the molten salt flow and the heat transfer rate of the paddle $\dot{Q}$), we used a finite-volume computational package ANSYS Fluent (14.5, ANSYS, Canonsburg, PA, USA) [28], with the pressure-based solver and SIMPLE algorithm for pressure–velocity coupling, and the second order upwind scheme for momentum and energy equations. The residuals for mass, momentum, turbulent kinetic energy and dissipation rate equations are 10^−4^, and for the energy equation, the residuals are 10^−6^. The mesh independence for each simulation was checked. A maximum of 1% changes in pressure drop and heat transfer rate between successive mesh sizes are considered acceptable results. The number of grids varies from case to case, from a few million to more than 10 million. An example of the mesh independence check is given in Table 3. In Table 3, for 7,539,242 and 6,825,664 elements, the differences of the pressure drop (30,997 Pa and 30,992 Pa) and heat transfer rate (2836 W and 2841 W) are both less than 1%. In this case, 7,539,242 elements were selected in the simulations to ensure the mesh independence of the results.

According to the second law of thermodynamics, the irreversible processes take place due to two factors, to be specific, fluid flow and heat transfer. The total irreversibility can be measured by the entropy generation rate. Based on the first and second laws of thermodynamics, we obtain the energy balance and entropy balance equations for the heat transfer process as follows [29,30]:

(9) $$dE/d\tau ={\dot{Q}}_{w}-\dot{Q}+\dot{m}\left(h_{in}-h_{out}\right)=0,$$

(10) $$dS/d\tau =\frac{{\dot{Q}}_{w}}{T_{w}}-\frac{\dot{Q}}{T_{o}}+\dot{m}\left(s_{in}-s_{out}\right)+{\dot{S}}_{g}=0.$$

The entropy generation rate is derived as:

(11a) $${\dot{S}}_{g}=\frac{\dot{Q}}{T_{o}}-\frac{\dot{Q}+\dot{m}\left(h_{out}-h_{in}\right)}{T_{w}}+\dot{m}\left(s_{out}-s_{in}\right),$$

(11b) $${\dot{S}}_{g}=\frac{\dot{Q}}{T_{o}}-\frac{\dot{Q}+\dot{m}c_{p}\left(T_{out}-T_{in}\right)}{T_{w}}+\dot{m}\left[c_{p}ln\left(\frac{T_{out}}{T_{in}}\right)+\frac{\alpha_{V}\Delta P}{\rho }\right],$$

In Equation (11b), the *ΔP* term corresponds to the fluid flow irreversibility (*ΔP*-induced entropy generation rate). On the right side of Equation (11b), the sum of the terms except the *ΔP* term is the heat transfer-induced entropy generation rate.

Based on the CFD model (i.e., Equations (1)–(8)), the pressure drop and heat transfer rate are first obtained. Then, the entropy generation rate is calculated through Equation (11a) or Equation (11b). To compare the performance of hollow paddles and solid paddles, the simulation results are summarized in dimensionless groups as follows:

(12) $$M=\frac{\dot{m}c_{p}}{\lambda L},$$

(13) $$Be=\frac{\Delta PL^{2}}{\mu \alpha },$$

(14) $$R=\frac{\dot{Q}}{{\dot{Q}}_{s}},$$

(15) $$S=\frac{{\dot{S}}_{g}}{{\dot{S}}_{gs}},$$

In Equation (14), if *R* > 1, the heat transfer performance of the hollow paddle is better than that of the solid paddle. In Equation (15), if *S* > 1, the total irreversibility in the heat transfer process of the hollow paddle is larger than that of the solid paddle. Because we use Case I as the reference design to define dimensionless groups and the focus is to reveal the advantages or disadvantages of hollow paddles compared to solid paddles, in Section 4, we will not list Case I as an independent subsection. For Case I and *h_o_* = 250 W/(m^2^K), when *H* = 46, 92 and 184 mm, $\dot{Q}$ = 760, 929 and 1021 W, respectively. For Case I and *H* = 92 mm, when *h_o_* = 50 and 150 W/(m^2^K), $\dot{Q}$ = 429 and 758 W, respectively. Therefore, based on the dimensional values for Case I and the dimensionless values documented in Section 4, dimensional results for hollow paddles can be easily obtained.

## 4. Results and Discussion

### 4.1. Case II

In product designs, the outer shape of the hollow paddle is determined by the shell-side material, and there exists a least thickness of the paddle wall (*t* = 3 mm in this paper) regarding the requirement of the mechanical strength. Compared to pure conduction in the solid paddle, both conduction and convection in the hollow paddle affect the heat transfer process. The fluid volume in the hollow paddle is a critical design parameter. The fluid volume ratio is defined as:

(16) $$\phi =\frac{V_{f}}{V},$$

where *φ* = 0 corresponds to the solid paddles. Table 4 shows the simulated cases with different *φ*. 

**Figure 4:**
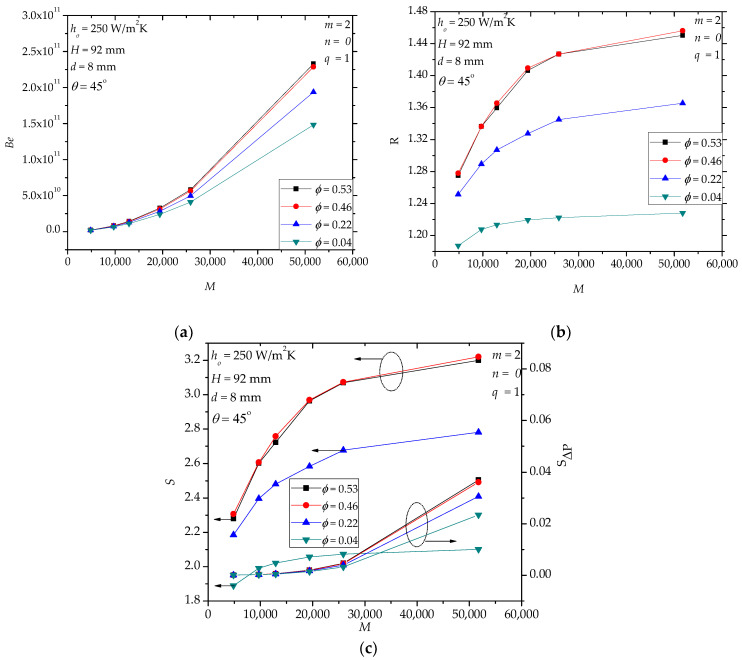
The effects of the fluid volume in hollow paddles. (**a**): *Be*; (**b**): *R*; (**c**): *S*, *S_ΔP_*.

**Figure 5:**
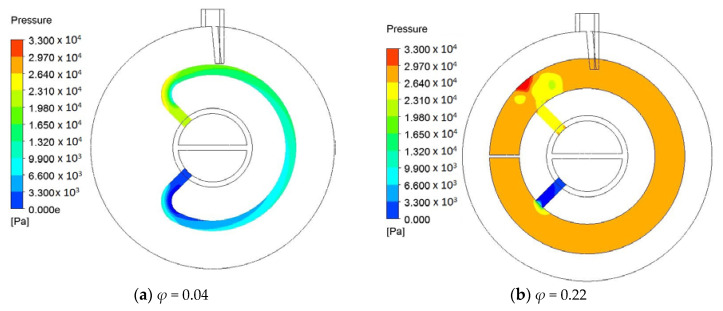

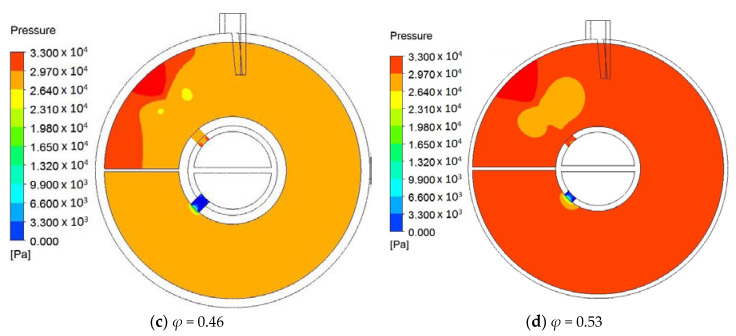
Pressure distribution (Case II, *m* = 2, *n* = 0, *q* = 1, *h_o_* = 250 W/m^2^K, *H* = 92 mm, *d* = 8 mm, *θ* = 45°, *M* = 25,881).

Figure 4 shows the effects of *φ* of hollow paddles. In Figure 4a, in the range *M* < 20,000, the effect of *φ* on *Be* is small. When *M* > 20,000, the difference of *Be* emerges for the designs with different *φ*. For specified *M*, *Be* increases with the increase in *φ*, especially when *φ* is small, e.g., *φ* = 0~0.46. For *φ* = 0.46 and 0.53, *Be* is nearly the same. Figure 5 shows an example of the pressure distribution in the middle cross-section of the fluid space. For the hollow paddle with *φ* = 0.04, the fluid space has a uniform cross-section area (i.e., constant diameter 8 mm), and for such flow in a tube, the pressure drop is mainly caused by the wall friction. Therefore, in this case, the distributed loss (depending on the diameter and the length of the tube for fixed mass flow rate and fluid properties) is dominant. However, for the hollow paddles with a large fluid space (e.g., *φ* = 0.22, 0.46 and 0.53), in sequence, the molten salt flows through the inlet flow hole with a small diameter, the internal space with both radial flow and circumferential flow and the outlet flow hole. This forms a complicated three-dimensional flow. Both the local losses (e.g., vortex, sudden-convergence and sudden-divergence) and the distributed losses are important. For some examples, like the case in Figure 5d, the pressure changes significantly near the outlet flow hole (sudden-divergence), which can be directly viewed from the color change. However, in other domains, the pressure does not change significantly. This observation tells us that the local loss near the outlet flow hole (sudden-divergence) is dominant.

In Figure 4b, all *R* values are greater than one, illustrating that the heat transfer performance of the hollow paddles is always better than the corresponding solid paddles in the specified range. In the range *φ* = 0~0.46, larger *φ* generates greater heat transfer rate because of stronger convection. This is the mechanism of the heat transfer enhancement of hollow paddles: the introduction of the convection reduces the thermal resistance of the conduction in the paddle. Figure 4b also shows that for *φ* = 0.46 and 0.53, there is no difference in *R*. This implies that the heat transfer improvement via increasing *φ* has an upper limit. In fact, larger *φ* leads to lower (average) fluid velocity in the fluid space, weakening the advantage of convection. This also means that a trade-off between conduction and convection exists. It is known that for a solid paddle, heat is transferred from its bottom surface (i.e., the hot surface) to its top surface and side surfaces through conduction. The thermal resistance of conduction is proportional to the heat transfer path length. When the hollow structure is introduced in the paddle, convection makes the temperature distribution in the hollow paddle more uniformly than pure conduction in the solid paddle. The effect of convection depends on the velocity. The larger the velocity, the less the thermal resistance. As the fluid volume increases, the velocity drops. If the velocity is too small, convection in the hollow paddle will not work. In Figure 4c, the total entropy generation rate (*S*) shows a similar trend as the heat transfer rate, which indicates that a greater heat transfer rate leads to larger irreversibility; the price for the heat transfer enhancement. The *ΔP*-induced entropy generation rate (*S_ΔP_*) increases with the increase of *M*, and it is much less than *S*, which means that the heat transfer contributes to the main irreversibility.

**Figure 6:**
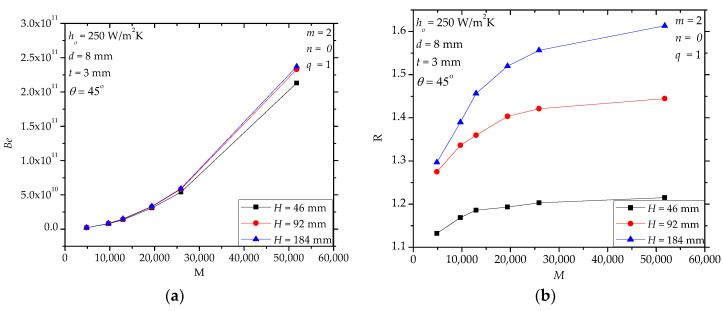

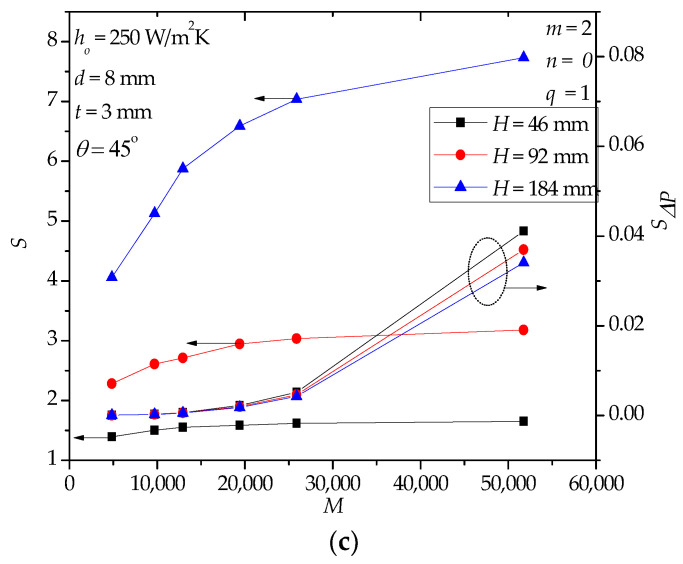
The effects of the paddle height (H). (**a**): *Be*; (**b**): *R*; (**c**): *S*, *S_ΔP_*.

For paddle heat exchangers, the paddle height *H* determines the heat transfer area and the material driving characteristics. Figure 6a shows that when *M* is small, e.g., less than 20,000, the effect of *H* on *Be* is negligible. When *M* becomes larger, *Be* of the design with *H* = 46 mm is less than that with *H* = 92 and 184 mm. Figure 6b shows that in the specified range, compared to the solid paddles, the improvement of the heat transfer performance of the hollow paddles is greater when *H* increases. This is understandable because larger *H* is equivalent to a longer path or larger thermal resistance of conduction. In this condition, hollow paddles are more favorable. Figure 6c further shows that *S* increases with the increase of *H* and the heat transfer-induced irreversibility is dominant.

**Figure 7:**
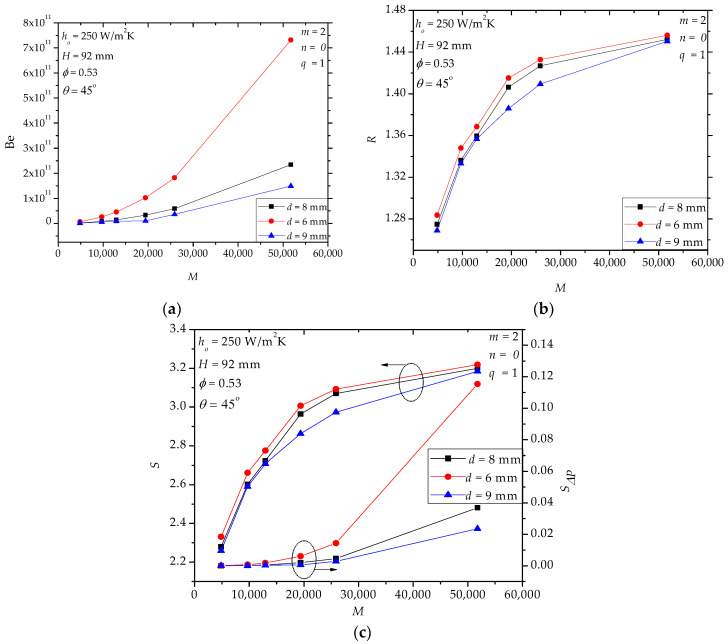
The effects of the diameter of the flow holes. (**a**): *Be*; (**b**): *R*; (**c**): *S*, *S_ΔP_*.

**Figure 8:**
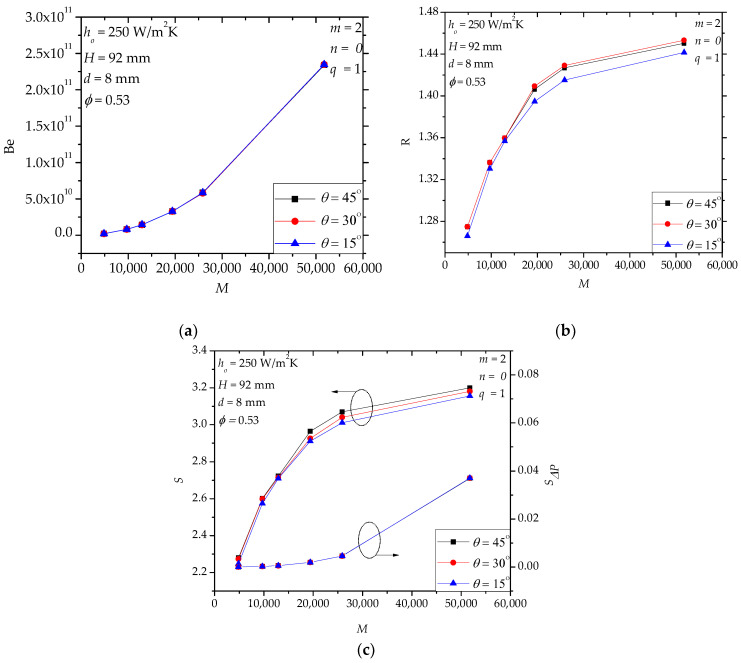
The effects of the position angle of the flow holes. (**a**): *Be*; (**b**): *R*; (**c**): *S*, *S_ΔP_*.

In hollow paddles, the diameter and the position angle of the flow holes influence the flow field and the stress distribution (or mechanical strength) of the shaft. Figure 7a shows that the diameter *d* affects *Be* significantly, especially when *M* is large, which means that the local loss of the molten salt flow is very important in the total pressure drop (also, cf. Figure 5d). Figure 7b shows that *d* has a weak effect on *R*. Actually, *d* only affects the local region near the inlet and outlet holes while the main heat transfer region (i.e., the internal fluid space) is nearly not influenced. In Figure 7c, when *d* decreases, *S_ΔP_*/*S* increases especially for large *M*. For example, when *M* = 51,756, *S_ΔP_*/*S*= 0.035, 0.011 and 0.007 for *d* = 6 mm, 8 mm and 9 mm, respectively. For the specified range in Figure 8, we notice that the effect of *θ* is weak. This observation encourages us to use larger *θ* to avoid stress concentration in the shaft. Actually, in Figure 8a (or Figure 8c), the curves of *Be* (or *S_ΔP_*/*S*) for different position angles overlap each other.

**Figure 9:**
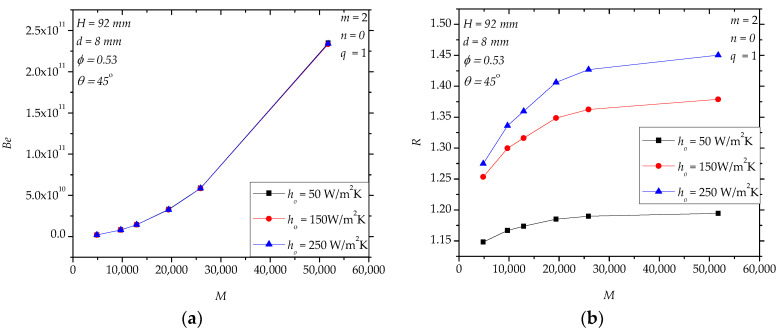

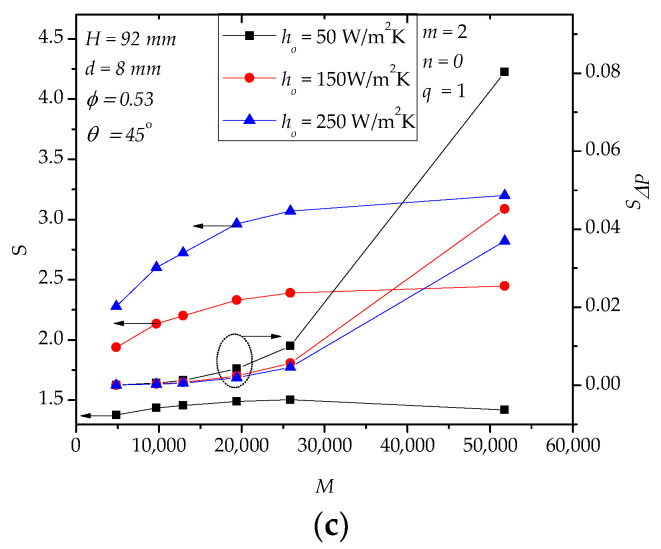
The effects of the shell-side material convective heat transfer coefficient (*h_o_*). (**a**): *Be*; (**b**): *R*; (**c**): *S*, *S_ΔP_*.

Although the shell-side material convective heat transfer coefficient (*h_o_*) does not affect *Be* (Figure 9a), it does influence *R* (Figure 9b). For a specified *M*, *R* increases with the increasing of *h_o_*. Even in the condition *h_o_* = 50 W/(m^2^K), *R* is greater than 1.15. In theory, when *h_o_* increases, the thermal resistance of conduction in the solid paddles becomes more dominant in which case hollow structures are more attractive. Because higher *h_o_* corresponds to a greater heat transfer rate, *S* is also larger (Figure 9c).

### 4.2. Case III

**Figure 10:**
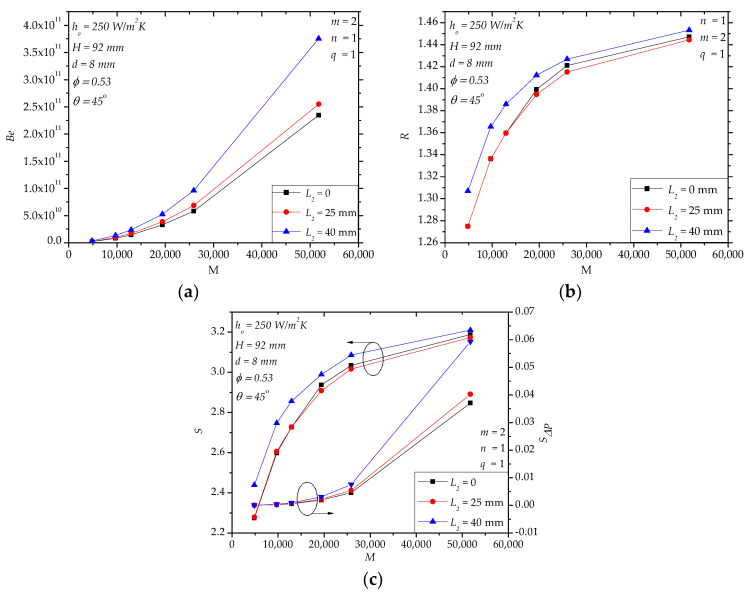
The effects of the geometry of the outlet baffle (*L*_2_). (**a**) *Be*; (**b**) *R*; (**c**) *S*, *S_ΔP_*.

In Section 4.1, we verified the goodness of hollow structures in enhancing the heat transfer of the paddles. In this section, we further explore the flow field modification through internal baffles, aiming to bathe the fluid space as uniformly as possible. One simple way is to introduce one baffle (*n* = 1) near the outlet of the fluid space in order to narrow the corner region with poor flow near the outlet of the fluid space [23]. Increasing the height of the baffle (*L*_2_) obviously leads to higher pressure drop when *M* > 20,000 (Figure 10a). For *L*_2_ = 0 (*n* = 0, no baffle) and *L*_2_ = 25 mm, *R* is very close in the specified range of *M*, and for *L*_2_ = 40 mm, *R* is slightly greater (Figure 10b). For example, for *M* = 4849, *R* with *L*_2_ = 40 mm is 2.5% higher than that of *L*_2_ = 0 mm. The entropy generation rate *S* in Figure 10c displays a similar trend as *R* in Figure 10b. For large *M* (e.g., *M* > 25,000), *Be* increases with the increasing in *L*_2_, so *S_ΔP_* increases, as well.

**Figure 11:**
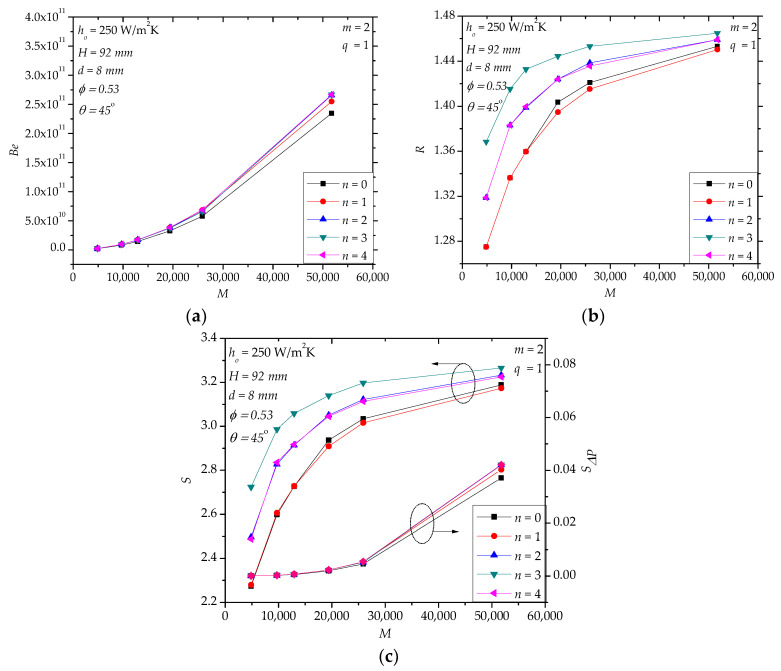
The effects of the number of baffles. (**a**) *Be*; (**b**) *R*; (**c**) *S*, *S_ΔP_*.

The effects of the number of the internal baffles (*n*) are shown in Figure 11. Here, we assume that the baffles are arranged uniformly and have a fixed height (*L*_2_ = 25 mm). In the range *M* < 20,000, *n* does not have a significant effect on *Be* (Figure 11a). When *M* > 20,000, there is limited gap in *Be* for *n* = 0 and *n* > 0. For *n* = 1~4, the gap of *Be* is small. From Figure 11b,c, we see that the effects of *n* are not monotonous. For example, for *M* = 9706, *R* for *n* = 3 is the largest and 6% greater than that for *n* = 0. For *M* = 9706, *S* for *n* = 3 is also the largest and 15% greater than that for *n* = 0. Realize that the present number of baffles (Figure 11) is few (*n* < 5). Much more baffles or three-dimensional baffles may generate plug flow that is good for convective heat transfer, but undoubtedly, the price is much higher pressure drop and manufacture cost.

### 4.3. Case IV

**Figure 12:**
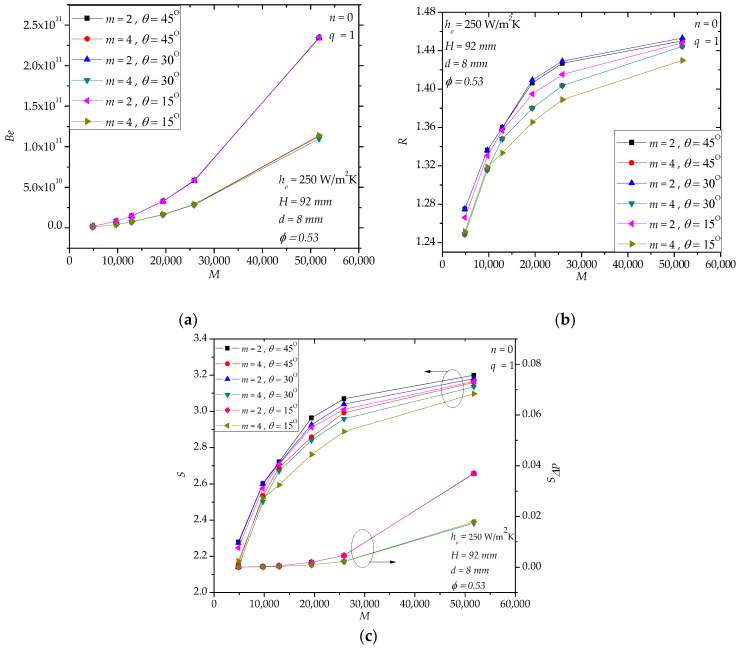
The effects of the number of the flow holes (m). (**a**) *Be*; (**b**) *R*; (**c**) *S*, *S_ΔP_*.

Figure 12 shows the simulation results of the four-hole design, i.e., two inlet flow holes and two outlet flow holes. Seen in Figure 12a, *Be* of the four-hole design is significantly less than that of the two-hole design in most of the specified range. When *M* increases, the gap between the two designs becomes larger. This is the obvious advantage of the four-hole design. However, *R* of the four-hole design reduces only 1.3~2.9% compared to that of the two-hole design in the specified range (Figure 12b). Note that Figure 12b is only a case study. For other conditions, the quantitative effect of the four-hole design may not be the same. Correspondingly, the entropy generation rate (*S*) of the four-hole design reduces 1.2~5.1% compared to that of the two-hole design in the specified range (Figure 12c). We know that the hollow paddles are welded on the outer surface of the hollow shaft, and the fluid space in the paddles is connected to that in the hollow shaft through the flow holes. More flow holes, corresponding to less pressure drop as shown in Figure 12, mean lower mechanical strength of the shaft. For sure, the four-hole design will weaken the mechanical strength of the shaft more heavily than the two-hole design, so the trade-off between the thermo-fluid design and the mechanical design is necessary. The analysis of the mechanical strength is beyond the scope of the present paper.

Theoretically, more holes (e.g., *n* = 6) is also possible. Note that in Figure 12a, c, the values of *Be* and *S_ΔP_*/*S* are approximately the same along the range, so the curves cover each other.

### 4.4. Further Discussion

**Figure 13:**
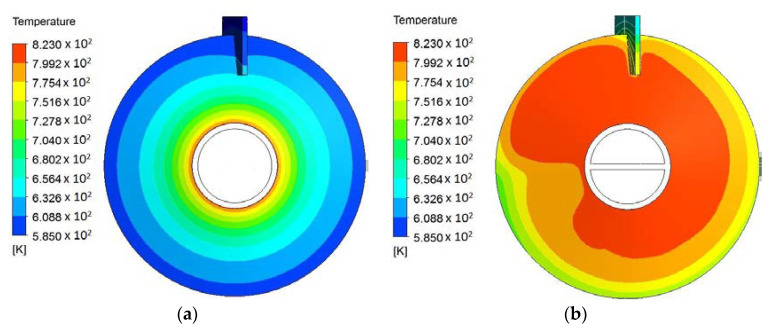
Temperature fields of outer surfaces: (**a**) solid paddle; (**b**) hollow paddle (*m* = 2, *n* = 0, *q* = 1, *h_o_* = 250 W/(m^2^K), *H* = 92 mm, *d* = 8 mm, *φ* = 0.53, *θ* = 45°, *M* = 25,881).

In the above sections, we showed the efficacy of using hollow paddles to enhance heat transfer. At the same time, the temperature uniformity of the paddles is also improved. Figure 13 provides an example of the temperature fields of the outer surfaces of two designs, namely one solid paddle (the reference design) and one hollow paddle. Apparently, the hollow paddle shows better temperature uniformity, and its average temperature approaches the inlet molten salt temperature more closely.

**Figure 14:**
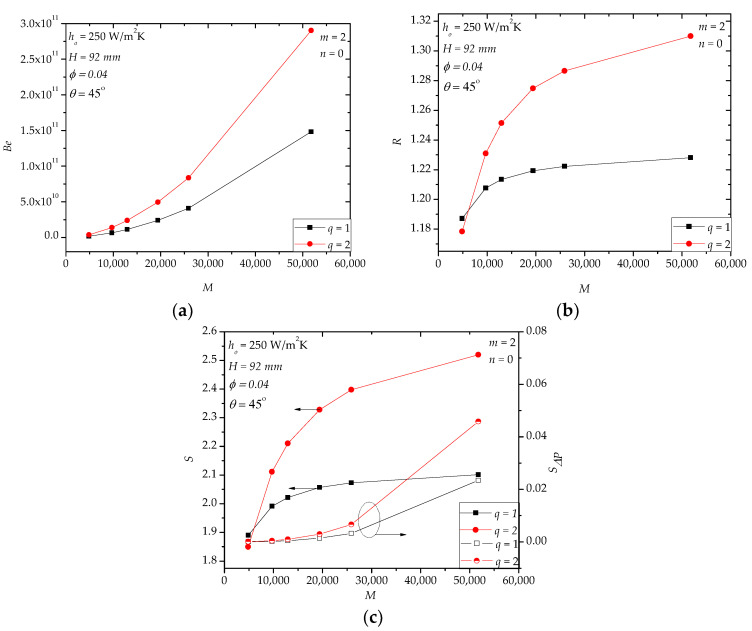
Two fluid channels in the paddles. (**a**) *Be*; (**b**) *R*; (**c**) *S*, *S_ΔP_*.

**Figure 15:**
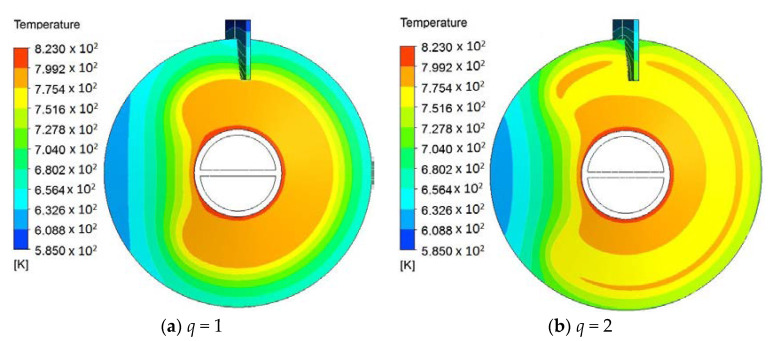
Temperature fields of outer surfaces for different channels (*m* = 2, *n* = 0, *h_o_* = 250 W/m^2^K, *H* = 92 mm, *d* = 8 mm, *φ* = 0.04, *θ* = 45°, *M* = 25,881).

For hollow paddles with a small fluid volume ratio (also called vascular structures [21]), e.g., *φ* = 0.04, an alternative way of further improving the heat transfer rate is to optimize the distribution of the fluid channels, such as using multiple channels or multi-scale channels. In Section 4.1, for *φ* = 0.04, we used one tube with a diameter of 8 mm to form the fluid space. Here, in Figure 14, an example with two channels (*q* = 2) for *φ* = 0.04 is shown. In Figure 14a, *Be* for *q* = 2 is larger than that for *q* = 1. This is because the diameter of the two channels is less than that with only one channel. When *M* > 9706, *R* for *q* = 2 is greater than that for *q* = 1. When *M* < 9706, the gap between the two designs is limited. The entropy generation rate (*S*) shows a similar trend as *R*. Figure 15 shows the temperature fields of the designs with different *q*. The temperature uniformity of the design with *q* = 2 is better than that with *q* = 1.

## 5. Conclusions

In this paper, based on the widely-accepted CFD technique for single-phase flow and heat transfer, we used three-dimensional numerical simulations to investigate the fluid flow and heat transfer characteristics of high temperature molten salt flowing in single-leaf type hollow paddles. First, we showed that the heat transfer rate of the hollow paddles is significantly greater than that of the solid paddles. The heat transfer enhancement of the hollow paddles is attributed to convection replacing conduction partly in the paddles. The price of the heat transfer enhancement is additional pressure drop and a larger total entropy generation rate. The heat transfer-induced irreversibility is much larger than the pressure drop-induced irreversibility under the studied conditions.

Secondly, the effects of the main geometrical parameters and working conditions were revealed. Increasing the volume of the fluid space helps enhance the heat transfer, but there exists an upper limit. For a larger height of the paddles, the hollow paddles are more favorable compared to the solid paddles. The diameter of the flow holes influences the pressure drop strongly, but does not affect the heat transfer rate significantly. In the studied range, the effects of the position angles of the flow holes are weak. A larger material-side heat transfer coefficient corresponds to greater enhancement of heat transfer.

Finally, we discussed other possibilities of modifying the fluid flow and heat transfer, like using internal baffles to organize the flow field, using a four-hole design to replace the two-hole design or adopting multiple channels instead of a single channel for designs with small fluid volume. When the number of baffles is few (*n* < 5), the effects are limited. More flow holes reduce the pressure drop obviously. For the hollow paddles with small fluid volume, it is possible to increase the heat transfer rate with more fluid channels.

The complexity of the flow in hollow paddles lies on its three dimensionality, both radial and circumferential. Both local losses and distributed losses are important. The design selection depends on the trade-off among three factors, namely fluid flow (pressure drop), heat transfer and mechanical strength, although the mechanical strength was not included in this paper.

The present work reveals the goodness and badness of hollow paddles compared to solid paddles, which can help us make a decision about which will be selected in the real design of molten salt paddle heat exchangers. The research identifies the important factors that influence the pressure drop, the heat transfer rate and the total entropy generation rate and deepens our understanding of the heat transfer process for hollow paddles, e.g., from the viewpoint of irreversibility. It provides useful data (in dimensionless form) for design. The information in this paper is important and necessary for product design, manufacture and testing. Experimental work will be next.

## Figures and Tables

**Table 1:** The boundary conditions.

Paddle Structure		Boundary Condition
Fluid	Inlet	$\dot{m}$ *T_in_* = 550 °C
	Outlet	*P_out_* = 0 Pa
Shell-side material	Shell-side material	*T_o_* = 300 °C, *h_o_* = 250 W/(m^2^K)
Solid	Inside surfaces of shaft	*T_w_* = 550 °C
	End-wall surfaces of shaft	Adiabatic

**Table 2:** Properties of molten salt (*T* = 550 °C) and stainless steel 316L (*T* = 20 °C) [27].

Property	*ρ* (kg/m^3^)	*c_p_* (J/(kg K))	*λ* (W/(m K))	*μ* (kg/(m s))
Molten salt	1944	1559.886	0.908	0.0012
Stainless steel 316L	8000	500	21.5	

**Table 3:** Mesh independence checking (Case II, $\dot{m}$ = 0.39 kg/s, *h_o_* = 250 W/(m^2^K), *m* = 2, *n* = 0, *q* = 1, *H* = 92 mm, *d* = 8 mm, *φ =* 0.53, *θ* = 45°).

**Number of elements**	7,539,242	6,825,664	6,094,076	5,003,249	4,004,098
**Number of nodes**	1,866,142	1,731,115	1,566,281	1,318,141	1,063,456
**Pressure drop *ΔP* (Pa)**	30,997	30,922	31,131	31,430	28,020
**Heat transfer rate** $\dot{Q}$ **(W)**	2836	2841	2848	2848	2942

**Table 4:** Cases of hollow paddles with different *φ* (*m* = 2, *n* = 0, *q* = 1, *H* = 92 mm, *d* = 8 mm, *θ* = 45°).

*φ*	Paddle Type	Geometry Description
0	Solid	Reference: no fluid space in the paddle.
0.04	Hollow	The fluid space is a tube of 8 mm diameter in the paddle.
0.22	Hollow	*t* = 20 mm
0.46	Hollow	*t* = 6 mm
0.53	Hollow	*t* = 3 mm

## Nomenclature

*Be*	Dimensionless pressure drop, i.e., Bejan number
*c_p_*	Specific heat at constant pressure, J/(kgK)
*C*_1ε_ *, C*_2ε_ *, C_µ_*	Constants, Equations (7) and (8)
*d*	Diameter of flow holes, mm or m
*e*	Specific total energy, J/kg
*E*	Total energy, J
*h*	Enthalpy, J/kg
*h_o_*	Shell-side material convective heat transfer coefficient, W/(m^2^K)
*H*	Paddle height, mm or m
*k*	Turbulent kinetic energy, m^2^/s^2^
*L, L*_1_ *, ..., L*_5_	Dimensions, mm or m, Figure 3
*m*	Number of flow holes
$\dot{m}$	Mass flow rate, kg/s
*M*	Dimensionless mass flow rate
*n*	Number of baffles
*P*	Pressure, Pa
*Pr_t_*	Turbulent Prandtl number
*q*	Number of channels
$\dot{Q}$	Heat transfer rate, W
*R*	Dimensionless heat transfer rate
*s*	Specific entropy, J/(kgK)
*S*	Dimensionless entropy generation rate
${\dot{S}}_{g}$	Entropy generation rate, W/K
*t*	Paddle thickness, mm or m
*T*	Temperature, K or °C
*T_o_*	Shell-side material temperature, K or °C
*u*	Velocity component, m/s
$u^{\prime }$	Fluctuating velocity component, m/s
*v*	Velocity, m/s
*V*	Volume of a paddle, m^3^
*V_f_*	Fluid volume in a paddle, m^3^
*x*	Coordinate component, mm or m
**Greek symbols**	
*α*	Thermal diffusivity, m^2^/s
$\alpha_{V}$	Thermal expansion coefficient, 1/K
*δ_ij_*	Unit tensor
*ΔP*	Pressure drop, Pa
*ε*	Dissipation rate, m^2^/s^3^
*θ*	Position angle of flow holes
*λ*	Thermal conductivity, W/(mK)
*µ*	Dynamic viscosity, kg/(ms)
*µ_t_*	Turbulent viscosity, kg/(ms)
*ρ*	Density, kg/m^3^
*σ_k_, σ_ε_*	Constants in Equations (6) and (7)
*τ*	Time
ϕ	Volume ratio, Equation (16)
**Subscripts**	
*i, j, l*	Coordinate direction
*in*	Inlet
*out*	Outlet
*s*	Solid
*w*	Wall
